# A case of TEVAR for aneurysmal re-expansion and hemoptysis post-FET

**DOI:** 10.1093/jscr/rjaf279

**Published:** 2025-05-03

**Authors:** Kotaro Mukasa, Yasunori Yakita, Musashi Tsuda, Shinichiro Abe, Soichi Asano

**Affiliations:** Department of Cardiovascular Surgery, Chiba Cardiovascular Center, 575 Tsurumai, Ichihara, Chiba 290-0512, Japan; Department of Cardiovascular Surgery, Chiba Cardiovascular Center, 575 Tsurumai, Ichihara, Chiba 290-0512, Japan; Department of Cardiovascular Surgery, Chiba Cardiovascular Center, 575 Tsurumai, Ichihara, Chiba 290-0512, Japan; Department of Cardiovascular Surgery, Chiba Cardiovascular Center, 575 Tsurumai, Ichihara, Chiba 290-0512, Japan; Department of Cardiovascular Surgery, Chiba Cardiovascular Center, 575 Tsurumai, Ichihara, Chiba 290-0512, Japan

**Keywords:** FET, TEVAR, endotension, migration, hemoptysis

## Abstract

This case study describes an 83-year-old man with a history of total arch replacement and frozen elephant trunk (FET) insertion for an aortic arch aneurysm presented with hemoptysis. Computed tomography revealed aneurysmal expansion; however, no obvious endoleaks were detected. The thoracic endovascular aortic repair was performed by deploying two stent grafts to cover the entire FET. Postoperatively, the hemoptysis resolved, and computed tomography revealed a reduction in the aneurysm size. Even in cases where no obvious contrast inflow is detected, it is necessary to suspect an occult endoleak if symptoms such as hemoptysis appear.

## Introduction

The frozen elephant trunk (FET) procedure is widely used for total arch replacement in patients with aortic arch aneurysms. However, it carries the risk of complications such as spinal cord injury, distal stent-induced new entry, and migration [1]. We report a case of hemoptysis due to aneurysmal re-expansion caused by an occult endoleak after FET insertion, which was successfully managed with thoracic endovascular aortic repair (TEVAR).

## Case report

**Figure 1:**
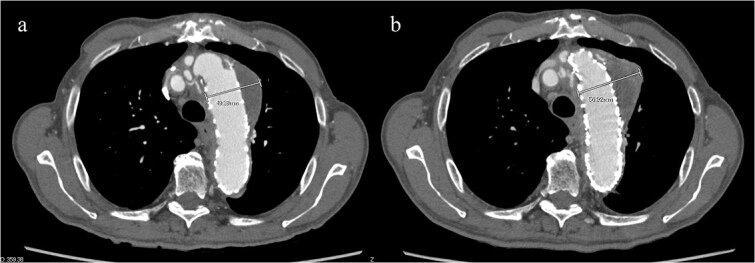
(a) Contrast-enhanced CT 1 year after total arch replacement with FET showing a reduction in aneurysm diameter from 55 mm to 49 mm, with no evidence of endoleak. (b) Contrast-enhanced CT 2 years after total arch replacement with a FET, showing an increase in aneurysm diameter to 56 mm.

An 83-year-old man presented at our hospital with intermittent hemoptysis. Two years before, he had undergone total arch replacement with FET insertion using a 29 × 120 mm Frozenix J graft (Japan Lifeline, Tokyo, Japan) for a 55 mm aortic arch aneurysm. The Frozenix J graft is a commercially produced open stent graft with an internal skeleton made of nickel-titanium alloy. One year after surgery, postoperative contrast-enhanced computed tomography (CT) showed a reduction in the aneurysm diameter to 49 mm, with no endoleaks (Fig. 1a).

**Figure 2:**
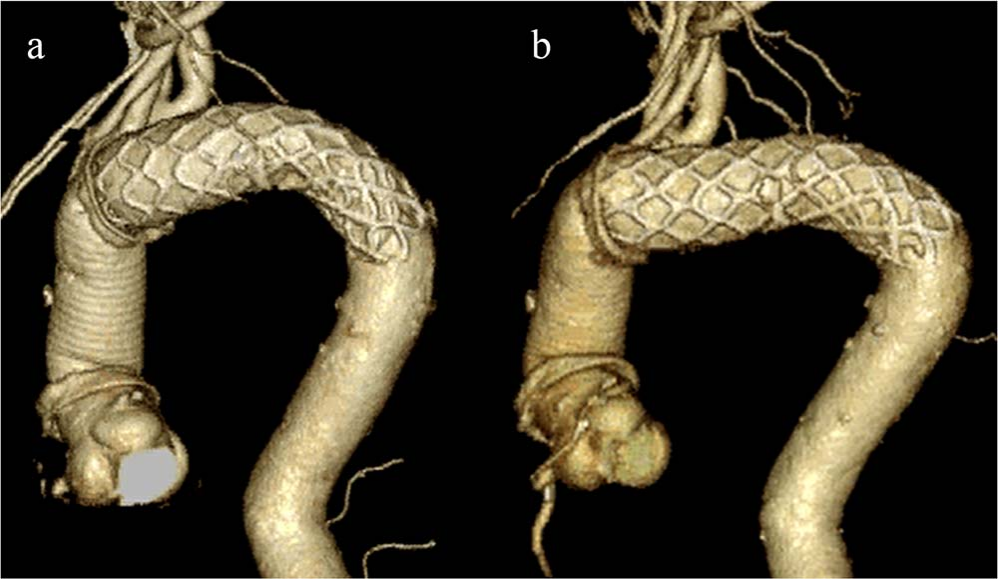
(a) Three-dimensional CT image 1 year after total arch replacement with a FET. (b) Three-dimensional CT image 2 years after total arch replacement with a FET demonstrating straightening and migration of the FET.

CT revealed no endoleak, but the aneurysm had enlarged to a diameter of 56 mm (Fig. 1b). Three-dimensional CT revealed straightening and proximal migration of the FET (Fig. 2). No clear signs of pulmonary parenchymal hemorrhage or findings suggestive of the mass were reported. Laboratory tests indicated mild anemia, with a hemoglobin of 9.7 g/dL and hematocrit of 29.0%. No infectious or inflammatory diseases that could cause hemoptysis were identified.

**Figure 3:**
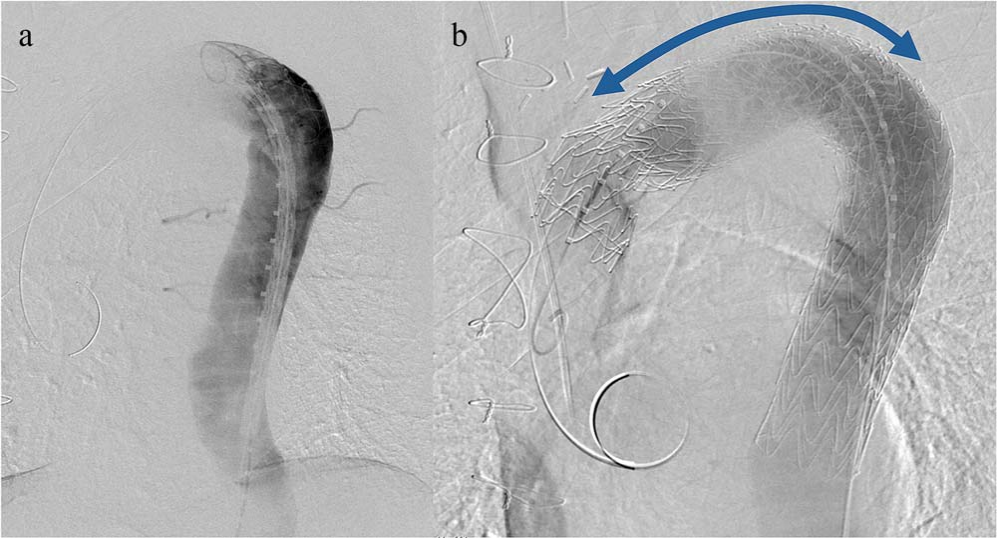
(a) Angiography performed from within the FET before deploying the stent graft showing no evidence of endoleak or aortobronchial fistula. (b) Angiography after stent graft placement. The bidirectional arrow shows the original extent of the FET insertion.

**Figure 4:**
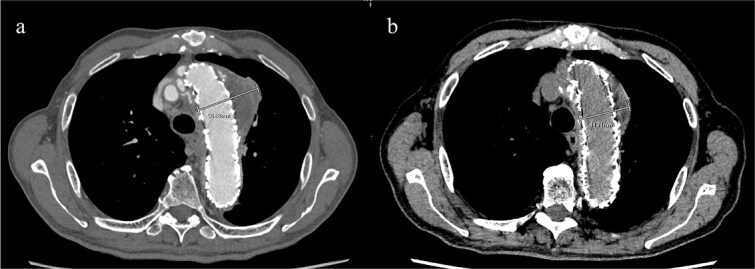
(a) Contrast-enhanced CT performed soon after TEVAR showing an aneurysm diameter of 56 mm. (b) Two years later, contrast-enhanced CT showing a reduction in the aneurysm diameter to 44 mm.

Given the ongoing hemoptysis, presumed aneurysmal expansion due to an occult endoleak, and the absence of any other obvious causes of hemoptysis, we determined that intervention for the aneurysm was necessary. TEVAR was performed using a right femoral artery approach. Initial angiography did not reveal contrast leakage into the sac (Fig. 3a). A 34 × 34 × 200 mm Gore Conformable TAG stent graft (W.L. Gore & Associates, Flagstaff, AZ, USA) was deployed to cover the distal edge of the FET. The second stent graft, a 34 × 34 × 150 mm Gore Conformable TAG stent graft, was deployed overlapping the first graft immediately after the left subclavian artery branch to cover the entire length of the FET (Fig. 3b). Considering the proximal migration, a type Ib endoleak was deemed the most likely cause. However, the possibility of a type III endoleak from the graft was also considered, so the stent graft was deployed to cover the entire area. The patient’s hemoptysis resolved completely postoperatively. A follow-up CT 2 years later showed a reduction in the aneurysm size from 56 to 44 mm (Fig. 4).

## Discussion

Endoleaks after FET have been reported in up to 28% of cases, with aneurysmal expansion occurring in 8% [2]. Despite the absence of visible endoleaks, aneurysmal expansion was observed. Many studies reported aneurysmal expansion without a detectable endoleak following endovascular repair of abdominal aortic aneurysms [3]. This suggests the presence of an occult endoleak, in which persistent pressure from an undetectable microendoleak on CT contributed to its expansion.

The FET procedure is associated with complications, such as spinal cord injury, distal stent-induced new entry, and stent graft migration. Proximal migration may have occurred because of the spring-back force. In this case, the occult endoleak may have been a subtle type Ib endoleak caused by the proximal migration of the distal edge. To prevent migration due to spring-back, in cases of fusiform aneurysms, it is desirable to deploy the stent graft along the greater curvature.

We planned TEVAR with the primary goal of extending the distal landing zone of the FET. However, type IIIb endoleaks from small tears in the graft could not be completely ruled out; therefore, in this TEVAR procedure, stent-grafts were deployed to cover the entire graft. Furthermore, rather than placing the proximal landing within the Frozenix, which has an internal stent skeleton, it may be better to place the proximal landing within the portion of the vascular graft proximal to the distal anastomosis to prevent an endoleak. When performing total arch replacement with FET insertion, we believe that it is necessary to maintain about 2 cm distance between the distal anastomosis and the left subclavian artery to ensure an adequate landing zone, considering the possibility of additional TEVAR.

Hemoptysis is a symptom of thoracic aortic aneurysms. Formation of an aortobronchial fistula can result in hemoptysis, and TEVAR is commonly employed as a treatment [4]. A report of recurrent hemoptysis due to a type II endoleak following TEVAR for an aortobronchial fistula has been published [5]. Despite the absence of clear CT findings of an endoleak or aortobronchial fistula, the patient presented with hemoptysis. Thoracotomy with pulmonary or aneurysm resection is often required to definitively identify or control the source of the microscopic alveolar hemorrhage. However, in older adult patients, as in this case, early TEVAR is crucial to prevent rupture and manage symptoms, even without definitive evidence of an endoleak. This is particularly important when there are findings suggestive of endotension, such as aneurysmal expansion or hemoptysis. This approach aligns with the overhaul concept used after EVAR [3].
